# Cardiac troponin I predicts clinical outcome of patients with cancer at emergency department

**DOI:** 10.1002/clc.23486

**Published:** 2020-10-21

**Authors:** Soo Hyun Park, Taerim Kim, Won Cul Cha, Hee Yoon, Sung Yeon Hwang, Tae Gun Shin, Min Seob Sim, IkJoon Jo, Seung‐Hwa Lee, Hyung‐Doo Park, Jin‐Ho Choi

**Affiliations:** ^1^ Department of Medicine, Graduate School Kyung Hee University Seoul South Korea; ^2^ Department of Emergency Medicine, Samsung Medical Center Sungkyunkwan University School of Medicine Seoul South Korea; ^3^ Department of Medicine Samsung Medical Center, Sungkyunkwan University School of Medicine Seoul South Korea; ^4^ Department of Laboratory Medicine and Genetics Samsung Medical Center, Sungkyunkwan University School of Medicine Seoul South Korea

**Keywords:** biomarker, cardiac troponin, cancer, emergency departments

## Abstract

**Background:**

The prognostic ability of cardiac troponin I (TnI) has been demonstrated in general populations and among cardiovascular disease patients, but it has not been evaluated in cancer patients.

**Hypothesis:**

This study assumes to have the prognostic ability of cardiac troponin in cancer patients visiting the emergency department.

**Methods:**

Cancer patients visiting the emergency department were enrolled in this retrospective cohort study. Patients with previously known coronary artery disease or clinically indicated coronary angiography were not included. The maximal value from Siemens ADVIA Centaur troponin I Ultra assay within 24 hours was assessed. The primary endpoint was 180‐day all‐cause death, including cardiovascular and noncardiovascular death.

**Results:**

A total of 9135 cancer patients (mean age: 63 years, male gender: 60%) were enrolled. Lowest (0.006 ng/mL), assay‐specific <99th % (0.007‐0.039 ng/mL), below median ≥ 99th % (0.040‐0.129 ng/mL), and above median ≥ 99th % (≥0.130 ng/mL) TnI were found in 4487 (49.1%), 3158 (34.6%), 852 (9.3%), and 638 (7.0%) patients, respectively. There was 3192 (34.9%) all‐cause deaths including 137 (1.5%) cardiovascular and 3047 (33.4%) noncardiovascular deaths in the 180‐day follow‐up period. The risks of all‐cause, cardiovascular, and noncardiovascular death increased across higher TnI strata (hazard ratio [HR] = 1.3‐2.9; 2.1‐9.3; 1.3‐1.8; *P* < .001, all). These findings were consistent within clinical subgroups including solid and hematologic cancers.

**Conclusions:**

Cancer patients visiting the emergency department with elevated troponin I were at increased risk of 180‐day death. Cancer patients with elevated TnI may need additional evaluation or careful follow‐up even without cardiovascular disease diagnosis.

## 1 INTRODUCTION

Cancer patients are increasingly visiting emergency departments.^1^ The emergency department is often the first place patients turn to when they have a complication or unexpected worsening of their condition. Cancer patients often require management of conditions caused by the cancer itself as well as the comorbidities and treatment effects. Emergency physicians frequently need to quickly evaluate patients' clinical needs, stratify individual risk, determine the most appropriate treatment, and decide whether to admit or provide outpatient care, all with limited time and resources. Case complexity can increase when patients present to the emergency department with little to no medical history information. A simple affordable risk‐predicting biomarker could be helpful in such situations.

Cardiac troponin is a highly sensitive and specific biomarker for myocardial infarction, and may be elevated in patients without clinically overt myocardial infarction.^2^ Cardiac troponin is frequently elevated in nonischemic cardiac conditions such as arrhythmias, heart failure, pericarditis, and also in noncardiac conditions such as pulmonary embolism, stroke, chronic kidney disease, sepsis or systemic inflammatory response syndrome (SIRS), critical illness, and drug‐causing cardiotoxicity.^3^ Although the pathophysiology of cardiac troponin elevation in nonischemic cardiac or extracardiac conditions are not well elucidated, elevated cardiac troponin showed prognostic implications not only in acute coronary syndrome but also in various clinical settings regarding patients with nondiagnosed chest pain and patients using statins for primary prevention.^4^, ^5^, ^6^, ^7^, ^8^

Therefore, we reasoned that troponin may predict clinical outcomes in patients with cancer. We investigated the ability of cardiac troponin to predict mid‐term (180 days) death risk in cancer patients who visited the emergency department of a tertiary hospital.

## 2 METHODS

### 2.1 Patients

We performed a retrospective single‐center cohort study of patients over 18 years old who visited the emergency department at Samsung Medical Center, a tertiary hospital located in Seoul, and who underwent a cardiac troponin I test within 24 hours of arrival between January 2007 and May 2016. Patients without a current or prior history of cancer were excluded. Patients with the following specific conditions that can accompany troponin elevation were also excluded: having a mechanical circulatory‐support device, ever underwent resuscitation, history of heart transplantation, coronary artery disease history, revascularization, or clinically indicated coronary angiography during hospital stay.

The Samsung Medical Center Institutional Review Board approved and waived the requirement for informed consent because only anonymized data were involved and results were reported in aggregate. This study followed the Strengthening the Reporting of Observational Studies in Epidemiology (STROBE) guidelines (supplementary document).

### 2.2 Data sources and definitions

All data were retrieved from the Clinical Data Warehouse Center at Samsung Medical Center (Darwin‐C). In cases of mortality outside the hospital, the specific cause of death was retrieved from the government operated organization Statistics of Korea. The timing of all clinical events, arrival or discharge time, death, and laboratory tests were defined using the timestamp in patients' electrical medical records. The study index date was used as the visit time to the emergency department.

The Siemens ADVIA Centaur XP analyzer (Munich, Germany) was used for cardiac troponin I measurement. It has a lowest cardiac troponin I (TnI) analytic sensitivity of 0.006 ng/mL, an upper refence limit at the 99th % of 0.040 ng/mL, and a coefficient of variation <10% at 0.030 ng/mL. The maximum level of TnI within 24 hours after emergency department visit was grouped into four categories using the lowest (0.006 ng/mL), <99th % (0.007‐0.039 ng/mL), less than median ≥ 99th % (0.040‐0.129 ng/mL), and median ≥ 99th % or higher (≥0.130 ng/mL). In instances of multiple TnI measurements, the highest value measured within 24 hours was selected. The first measured value was used for the other laboratory tests and vital sign measurements. All electrocardiography ST‐T changes were independently read and confirmed by experienced physicians (M.S. and J.C.).

The primary endpoint was 180‐day all‐cause death. The major cause of death was determined by the death codes in the 10th revision of the International Statistical Classification of Diseases and Related Health Problems (ICD‐10) and it was used to classify cardiac and noncardiac deaths (supplementary document). No patient was lost to follow‐up with respect to death.

### 2.3 Statistical analyses

Clinical characteristics are shown according to TnI levels. Continuous data are described as means with standard deviations. Categorical data are presented as frequencies. Differences were assessed with the Kruskal‐Wallis or chi‐square test. Changing trends across TnI levels were assessed with the Jonckheere‐Terpstra test.

Associations between TnI level and death were assessed with Cox regression models. For sensitivity analysis, three multivariate Cox regression models adjusted for the following clinical parameters were used: model 1 included basic demographics and visit year; model 2 included model‐1 parameters and clinical comorbidities and dyspnea symptoms, which were the most associated symptoms among TnI level and have been shown to have prognostic value in cancer patients visiting emergency departments^9^; model 3 included model‐1 and model‐2 parameters and electrocardiographic change, vital signs, and laboratory tests.

R version 3.6 (R Foundation for Statistical Computing, Vienna, Austria) was used for analyses. Hazard ratios (HR) for compared outcomes are reported with a 95% confidence interval (CI). Statistical significance was defined by two‐tailed *P* < .05.

## 3 RESULTS

### 3.1 Study population

**FIGURE 1:**
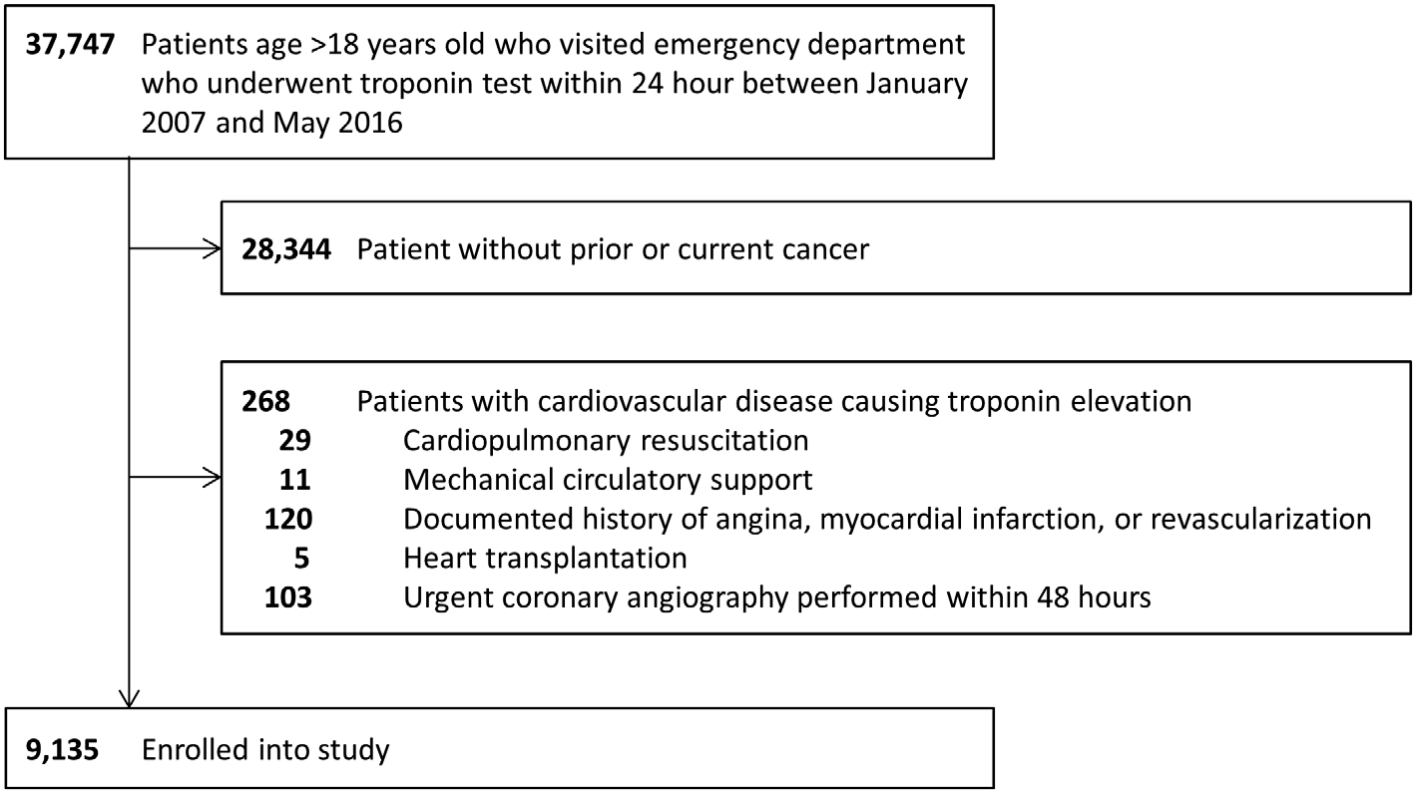
Flowchart shows inclusion and exclusion criteria for selecting patients to enroll into the study

A total of 37 747 patients who visited the emergency department and underwent TnI testing within 24 hours were screened for potential cardiovascular disease. Most TnI results were available within 2 hours (median: 1.1, interquartile range: 0.8‐1.9 hours). After excluding 28 344 patients without cancer and 268 patients who underwent cardiopulmonary resuscitation, received mechanical circulatory support, had a prior history of coronary artery disease diagnosis or revascularization, or heart transplantation, or underwent urgent coronary angiography within 48 hours of visiting the emergency department, a total of 9135 patients were enrolled in this study (Figure 1).

### 3.2 Clinical presentation

The number of patients with TnI = 0.006 ng/mL, 0.007‐0.039 ng/mL, 0.040‐0.129 ng/mL, and ≥ 0.130 ng/mL were 4487 (49.1%), 3158 (34.6%), 852 (9.3%), and 638 (7.0%), respectively. Age, male gender, and most clinical risk comorbidities increased with higher TnI levels. Typical angina‐like chest pain did not increase with TnI but dyspnea and other respiratory symptoms did (*P* < .001, Table 1; Supplementary Figure 1).

**TABLE 1:** Clinical characteristics

	TnI = 0.006 ng/mL (n = 4487)	TnI = 0.007‐0.039 ng/mL (n = 3158)	TnI = 0.040‐0.129 ng/mL (n = 852)	TnI ≥0.130 ng/mL (n = 638)	*P*value
Age	59.6 ± 12.6	65.4 ± 12.4	65.6 ± 13.3	66.5 ± 12.5	<.001
Age ≥ 65 years	1654 (36.9)	1862 (59.0)	493 (57.9)	386 (60.5)	<.001
Male gender	2585 (57.6)	1995 (63.2)	518 (60.8)	375 (58.8)	<.001
Diabetes	446 (9.9)	440 (13.9)	135 (15.8)	98 (15.4)	<.001
Hypertension	1157 (25.8)	1202 (38.1)	348 (40.8)	255 (40.0)	<.001
Respiratory disease	431 (9.6)	394 (12.5)	106 (12.4)	75 (11.8)	<.001
Hepatic disease	521 (11.6)	367 (11.6)	111 (13.0)	85 (13.3)	.42
Solid cancer	4273 (95.2)	2922 (92.5)	773 (90.7)	578 (90.6)	<.001
Hematologic cancer	574 (12.8)	515 (16.3)	156 (18.3)	134 (21.0)	<.001
Systolic blood pressure (mmHg)	124 ± 25	124 ± 29	121 ± 31	118 ± 32	<.001
Diastolic blood pressure (mmHg)	77 ± 15	76 ± 16	74 ± 17	72 ± 18	<.001
Heart rate (/min)	92 ± 22	97 ± 24	104 ± 27)	104 ± 27	<.001
Body mass index (kg/m^2^)	22.43 ± 3.52	22.54 ± 3.65	22.12 ± 3.81	22.16 ± 3.55	.009
Electrocardiography available	3063 (68.3)	2444 (77.4)	671 (78.8)	500 (78.4)	<.001
Electrocardiography ST‐T change	235 (7.7)	373 (15.3)	151 (22.5)	156 (31.2)	<.001
Symptom					<.001
Angina‐like chest pain	106 (2.4)	48 (1.5)	6 (0.7)	9 (1.4)	
Atypical chest pain	227 (5.1)	154 (4.9)	24 (2.8)	43 (6.7)	
Body pain other than chest	54 (1.2)	31 (1.0)	5 (0.6)	3 (0.5)	
Syncope or palpitation	296 (6.6)	187 (5.9)	44 (5.2)	23 (3.6)	
Neurologic symptom	258 (5.7)	188 (6.0)	81 (9.5)	73 (11.4)	
Dyspnea or respiratory symptom	972 (21.7)	797 (25.2)	272 (31.9)	216 (33.9)	
Gastrointestinal symptom	853 (19.0)	465 (14.7)	113 (13.3)	49 (7.7)	
Fever	490 (10.9)	433 (13.7)	131 (15.4)	95 (14.9)	
Dysuria or urologic symptom	23 (0.5)	27 (0.9)	5 (0.6)	3 (0.5)	
Trauma or superficial bleeding	24 (0.5)	17 (0.5)	7 (0.8)	2 (0.3)	
General weakness	127 (2.8)	117 (3.7)	39 (4.6)	24 (3.8)	
Miscellaneous	6 (0.1)	1 (0.0)	0 (0.0)	1 (0.2)	
Not described	1051 (23.4)	693 (21.9)	125 (14.7)	97 (15.2)	
Laboratory results					
White blood cell (/mm^3^)	8490 ± 7390	8970 ± 9100	10 000 ± 10 210	10 520 ± 7680	<.001
Hemoglobin (g/dl)	11.8 ± 2.3	11.6 ± 2.4	11.2 ± 2.5	11.4 ± 2.2	<.001
Platelet (10^3^/mm^3^)	215.0 ± 105.7	203.1 ± 106.2	179.5 ± 104.4	183.7 ± 104.9	<.001
Blood urea nitrogen (mg/dl)	16.9 ± 11.5	22.6 ± 17.3	29.7 ± 24.3	30.8 ± 25.4	<.001
Creatinine (mg/dl)	0.88 ± 0.74	1.22 ± 1.33	1.61 ± 1.84	1.78 ± 2.01	<.001
eGFR (ml/1.73m^2^/min)	96.6 ± 35.7	80.4 ± 40.1	68.2 ± 37.9	64.2 ± 40.8	<.001

### 3.3 Clinical outcomes

There was a total of 3192 (34.9%) all‐cause deaths including 281 (3.1%) cardiac deaths and 2911 (31.9%) noncardiac deaths during the 180‐day follow‐up period. The proportion of cardiac deaths among all‐cause deaths increased across the higher TnI levels (5.4%‐17.2%, *P* < .001; Table 2, supplementary Figure 2).

**TABLE 2:** Clinical outcome

	TnI = 0.006 ng/mL (n = 4487)	TnI = 0.007‐0.039 ng/mL (n = 3158)	TnI = 0.040‐0.129 ng/mL (n = 852)	TnI ≥0.130 ng/mL (n = 638)	*P* value
Death within 180 days					
All‐cause death	1325 (29.5)	1161 (36.8)	403 (47.3)	303 (47.5)	<.001
Cardiovascular death	71 (1.6)	104 (3.3)	54 (6.3)	52 (8.2)	<.001
Noncardiac death	1254 (27.9)	1057 (33.5)	349 (41.0)	251 (39.3)	<.001
Cause of death					<.001
Cardiovascular disease	71 (5.4)	104 (9.0)	54 (13.4)	52 (17.2)	
Gastrointestinal disease	7 (0.5)	14 (1.2)	4 (1.0)	2 (0.7)	
Infection	5 (0.4)	10 (0.9)	5 (1.2)	6 (2.0)	
Respiratory disease	9 (0.7)	29 (2.5)	12 (3.0)	14 (4.6)	
Cancer	1230 (92.8)	998 (86.0)	326 (80.9)	227 (74.9)	
Neurological disease	1 (0.1)	2 (0.2)	0 (0.0)	1 (0.3)	
Miscellaneous	0 (0.0)	4 (0.3)	1 (0.2)	1 (0.3)	
Trauma	2 (0.2)	0 (0.0)	1 (0.2)	0 (0.0)	

**FIGURE 2:**
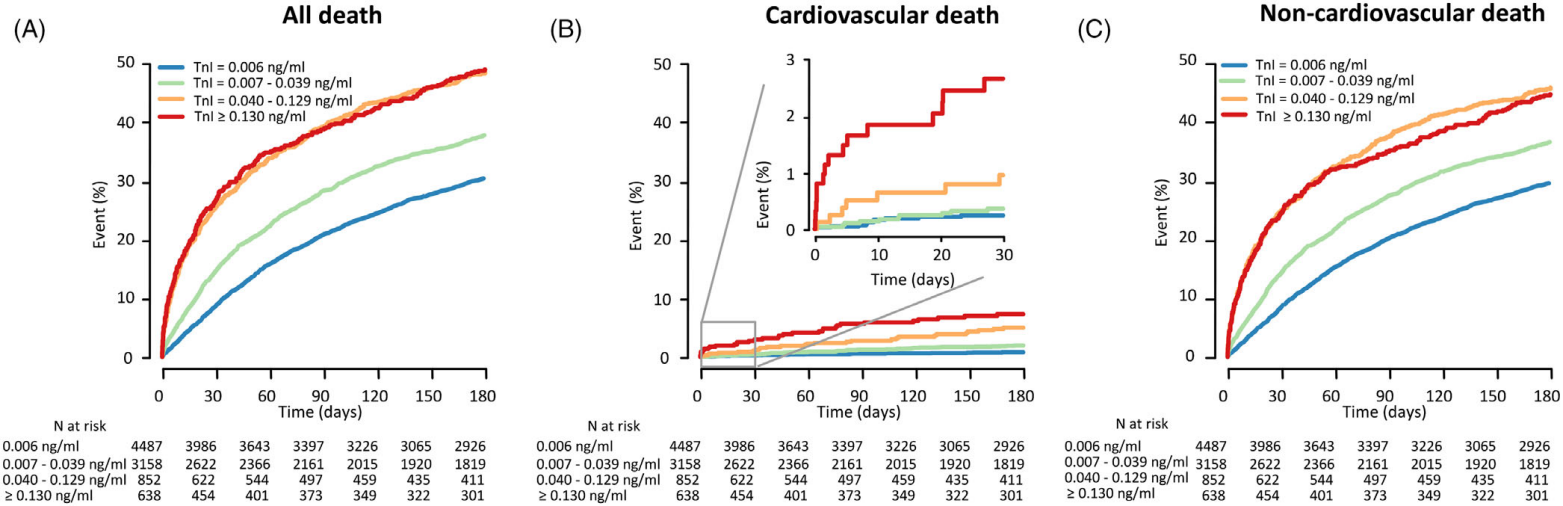
Panel A, B, and C shows that all the unadjusted 180‐day risks of all‐cause death, cardiovascular death, and noncardiovascular death increased across strata with higher TnI levels. See supplementary Table 1 for numerical data

The risks of all‐cause death and noncardiovascular death increased with higher TnI levels (HR = 1.34‐1.99, 1.32‐1.82, respectively, *P* < .001 for both), but there was no further increase of risk when TnI was higher than median ≥ 99 % (≥0.130 ng/mL). The risk of cardiovascular death increased consistently across higher TnI levels (HR = 2.08‐9.30, *P* < .001; Table 2, Figure 2).

**FIGURE 3:**
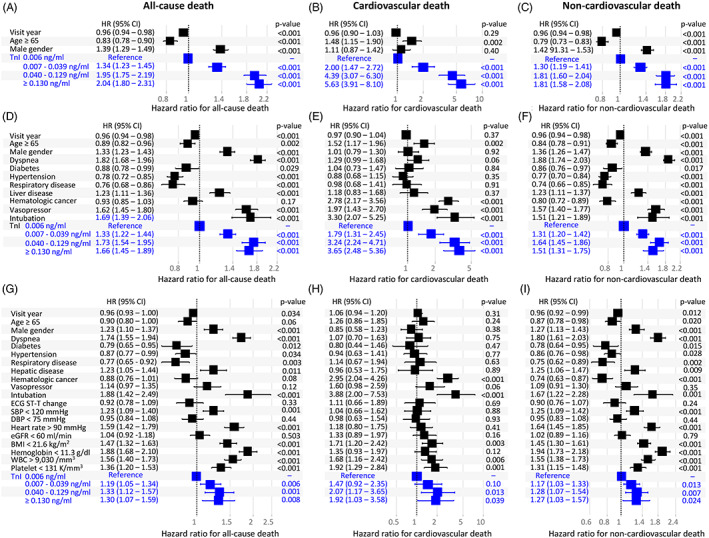
Panels A ‐ C show Cox regression models using demographics and visiting years. Panels D ‐ F show Cox regression models using clinical parameters in addition to parameters used in Panels A ‐ C. Panels G ‐ I show Cox regression models using laboratory results, vital signs, and ECG in addition to parameters used in Panels D ‐ F. TnI levels and associated data are shown in blue color. TnI, cardiac troponin I; ECG, electrocardiography; SBP, systolic blood pressure; DBP, diastolic blood pressure; eGFR, estimated glomerular filtration rate; BMI, body mass index; WBC, white blood cell

A set of multivariate Cox regression models showed that elevated TnI levels were independently associated with cardiac and noncardiac death as well as all‐cause death, not only in the basic model but also in the models that included clinical comorbidities, electrocardiographic change, and laboratory results (HR = 1.19‐5.63; *P* < .05 for all, except TnI = 0.007‐0.039 ng/mL for cardiac death; Figure 3).

## 4 DISCUSSION

In this retrospective cohort study, elevated TnI was independently associated with higher risk for both cardiac and noncardiac death in cancer patients visiting the emergency department who had not had a previous diagnosis of specific cardiac disease. Higher TnI levels were associated with increasing death risk. These results remained after sensitivity analyses using various multivariate Cox regression models. To the best of our knowledge, our study is the first to enroll a large number of patients and show prognostic ability of cardiac troponin in cancer patients visiting the emergency department.

Cardiovascular disease and cancer are the top two leading causes of death in industrialized countries and they share several common risk factors. Cancer patients are typically older and are likely to have multiple chronic diseases or comorbidities.^10^ These chronic disease or comorbidities may be preexisting or exacerbated by cancer progression or treatment, all of which complicate the clinical picture for prognostication in the emergency department. Our study subjects consisted of mid‐term high‐risk patients with an average 180‐day death risk of 34.9%. For such high‐risk populations, the decision to refer cancer patients to palliative care or to a hospice program is often required but is difficult to make. Prior studies have reported on many different potential prognostic factors, including disease progression, dyspneic symptoms, performance scores, functional status evaluated by hand‐grip strength, and various biomarkers including lactate dehydrogenase and albumin.^9^, ^11^ Use of cardiac troponin I for prognostication may help physicians make assessments for medical management of patients with cancer‐related complications or comorbidities.^12^

Our study showed associations but not causal relationships among elevated cardiac troponin I and clinical outcomes. Interestingly, the risk for noncardiac death was much higher than for cardiac death. This suggests that the risk of death might be driven mostly by patient comorbidities rather than by complications of myocardial ischemia or necrosis (Table 2).

Cardiac troponin level has predictive value but it was not compared with other parameters or predicting algorithms because of a lack of standardized guidelines to assess patients visiting the emergency department. Cancer patients visiting emergency departments comprise a highly heterogenous clinical picture and they potentially need individualized treatment and care. In situations with limited resources or time, cardiac troponin may be included in risk predicting algorithms and, as such, may reduce the knowledge gaps in clinical guidelines and operational challenges.

Cardiac troponin is an organ‐specific, not disease‐specific, biomarker. Although noncanonical expression of cardiac troponin in human cancer cells has been reported,^13^, ^14^ it is widely accepted that cardiac troponin is highly specific and sensitive to myocardial injury. However, elevation of cardiac troponin in cases of nonischemic cardiac disease or extracardiac disease is not uncommon.^15^ Elevation of cardiac troponin in cancer patients can be caused by myocardial ischemia secondary to reduced oxygen supply or increased oxygen demand, or by direct cardiac damage secondary to chemotherapy or infiltrative cardiac diseases.^16^ Chemotherapy, especially, can cause serious cardiovascular complications such as left ventricular dysfunction with heart failure, myocarditis, pericarditis, arrhythmia, or thromboembolism.^17^, ^18^ Anthracyclines, including doxorubicin and idarubicin, alkylating agents including cyclophosphamide and ifosphamide, targeted cancer therapy drugs including trastuzumab, bevacizumab, sunitinib, sorafenib, and carfilzomib, and recently introduced immune checkpoint inhibitors are widely recognized potential cardiotoxic agents.^17^, ^18^, ^19^ A meta‐analysis confirmed that abnormal troponin elevation predicted left ventricular dysfunction in patients receiving chemotherapy.^20^ Therefore, assessment of troponin for cancer patients may work as a screening tool to identify patients who could benefit from specific preventive strategies and may also enable early identification, evaluation, and cardiotoxicity monitoring during chemotherapy.^21^, ^22^, ^23^ Currently, recommendations surrounding the role of troponin for cancer patients are still based on expert consensus opinion. Further research with appropriately designed prospective trials is required to determine the optimal strategy for troponin testing in clinical practice.

### 4.1 Limitations

Although we enrolled any person who visited the emergency department to minimize selection bias, our study is not free from the inherent limitations of a retrospective single‐center study. Troponin testing was performed at the physician's discretion. There were no pre‐specified indications for troponin testing. The source data were administrative claims, which lack codes for specific conditions. We did not include patients who underwent coronary angiography during their hospital stay and the cause of TnI elevation was not investigated. The type, biology, and treatment setting for each patient's cancer were not investigated. The severity of chemotherapy‐ or radiation‐induced cardiotoxicity was not assessed.

### 4.2 Perspectives

#### 4.2.1 Medical knowledge competency

Cancer patients with elevated TnI in the emergency department had increased risk of 180‐day death.

#### 4.2.2 Patient care competency

It is important for emergency physicians, cardiologists, and oncologists to know that cancer patients with higher TnI levels have increased risk of death, which may help them make efficient and effective collaborative decisions about their healthcare strategy regarding aggressive, palliative, or hospice care given the available medical resources in crowded emergency departments.

#### 4.2.3 Translational outlook

Cancer patients usually have complex clinical conditions and comorbidities, and additional studies to design complex algorithms that include various predictors including TnI are needed to make better prognoses for life expectancy.

## DATA AVAILABILITY

The data that support the findings of this study are available on request from the corresponding author. The data are not publicly available due to privacy or ethical restrictions.

## Supporting information


**Supplementary Figure 1 Frequency of chief complaining symptom or electrocardiographic changes according to the cardiac troponin I (TnI) level**
Panel A: Frequency of chief complaining symptom according to the level of TnI. The frequency of dyspneic symptom increased across strata of higher TnI.
Panel B: Frequency of ECG ST‐T change according to the level of TnI, which increased across strata of higher TnI.

**Supplementary Figure 2** The increase of all‐cause death risk across cardiac troponin I (TnI) strata is mostly driven by the increase of cardiovascular death.

**Supplementary Table 1** Clinical outcome according to the cardiac troponin I (TnI) level
