# Supplementary figures and images for: Cardiac troponin I predicts clinical outcome of patients with cancer at emergency department
Source: Clin Cardiol. 2020 Oct 21;43(12):1585–91. doi: 10.1002/clc.23486 (PMC7724208; doi:10.1002/clc.23486)

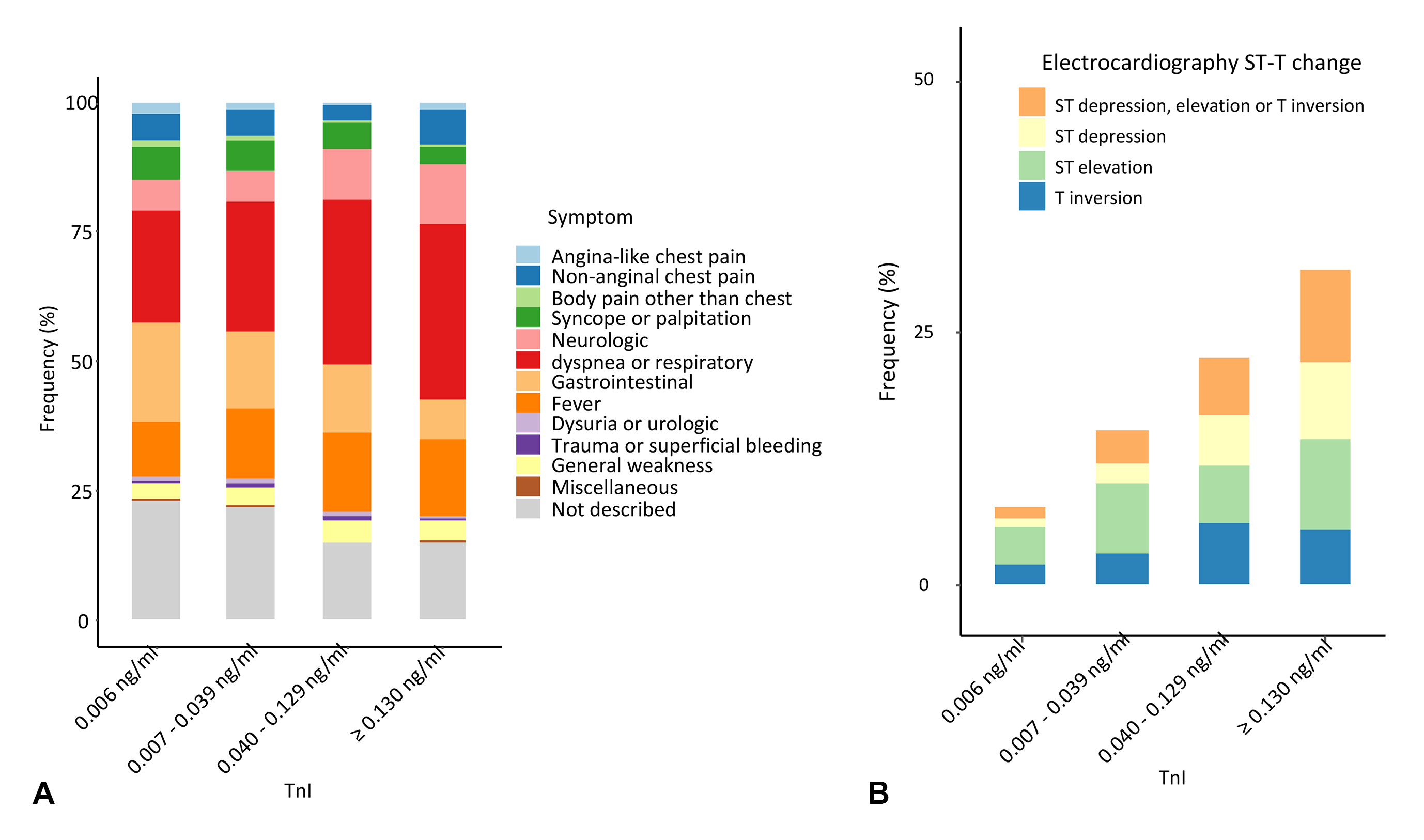

Supplement: Supplementary file 1 — Supplementary Figure 1 Frequency of chief complaining symptom or electrocardiographic changes according to the cardiac troponin I (TnI) level Panel A: Frequency of chief complaining symptom according to the level of TnI. The frequency of dyspneic symptom increased across strata of higher TnI. Panel B: Frequency of ECG ST‐T change according to the level of TnI, which increased across strata of higher TnI. [file CLC-43-1585-s001.tif]

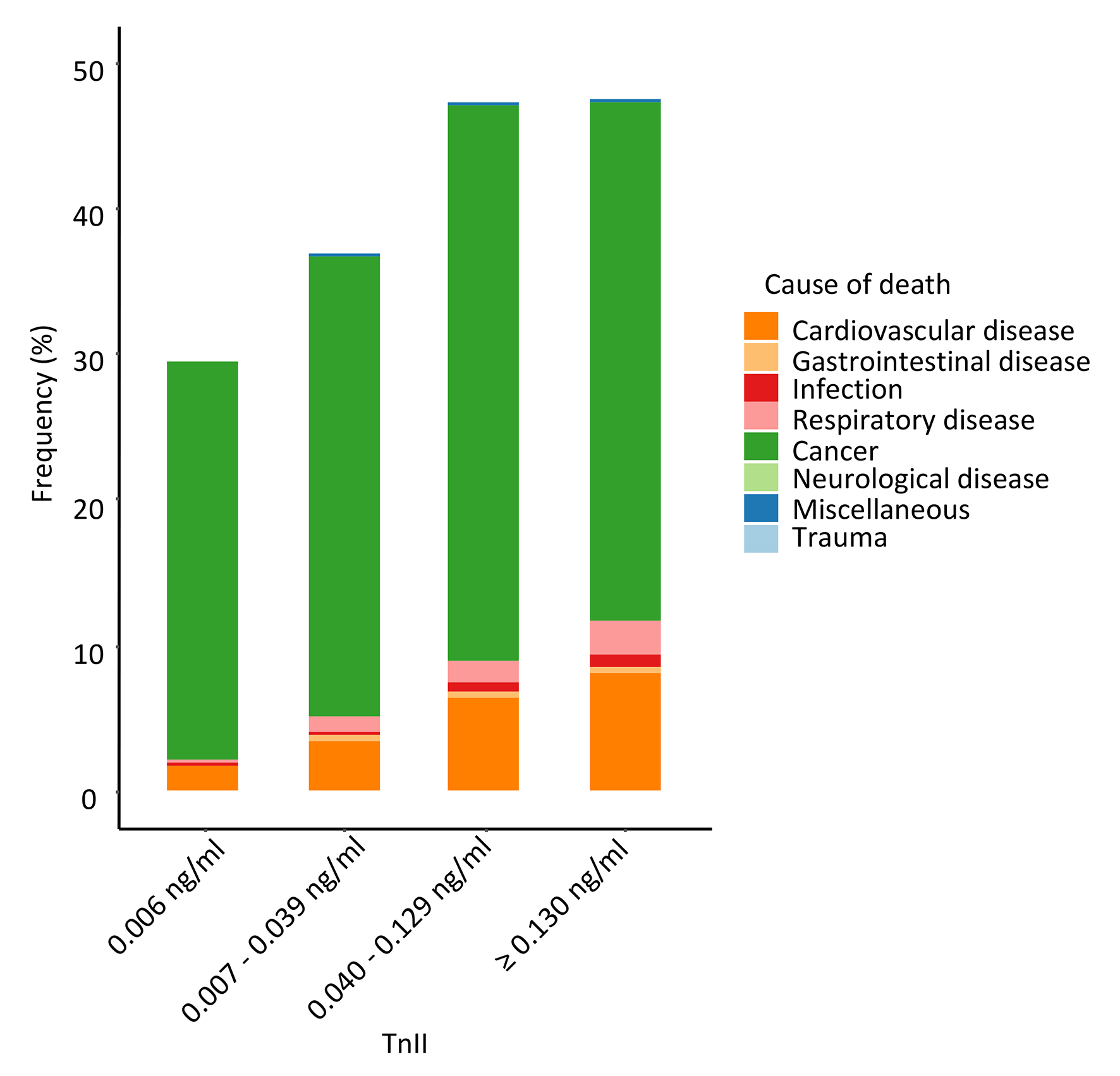

Supplement: Supplementary file 2 — Supplementary Figure 2 The increase of all‐cause death risk across cardiac troponin I (TnI) strata is mostly driven by the increase of cardiovascular death. [file CLC-43-1585-s002.tif]
