# Supplementary material for: Cardiac troponin I predicts clinical outcome of patients with cancer at emergency department
Source: Clin Cardiol. 2020 Oct 21;43(12):1585–91. doi: 10.1002/clc.23486 (PMC7724208; doi:10.1002/clc.23486)
Supplement: Supplementary file 3 — Supplementary Table 1 Clinical outcome according to the cardiac troponin I (TnI) level [file CLC-43-1585-s003.docx]

**Supplementary data
Supplementary Figure 1. Frequency of chief complaining symptom or electrocardiographic changes according to the cardiac troponin I (TnI) level**


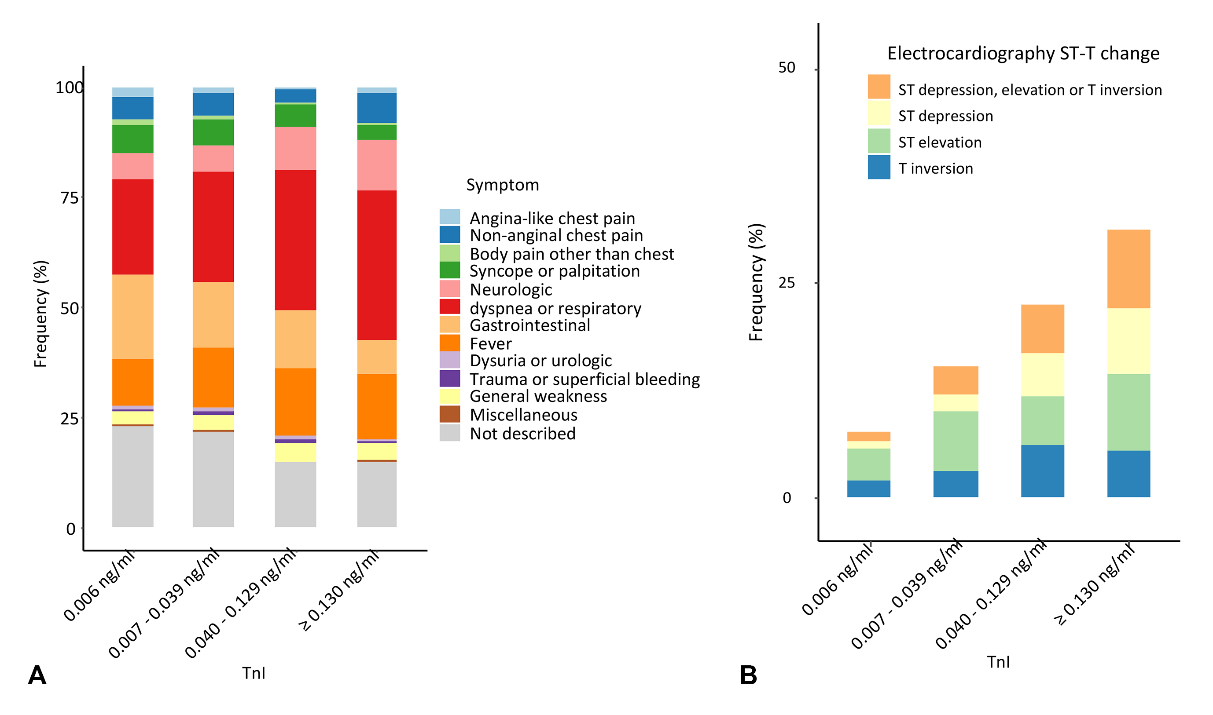

Panel A: Frequency of chief complaining symptom according to the level of TnI. The frequency of dyspneic symptom increased across strata of higher TnI.
Panel B: Frequency of ECG ST-T change according to the level of TnI, which increased across strata of higher TnI.

**Supplementary Figure 2** The increase of all-cause death risk across cardiac troponin I (TnI) strata is mostly driven by the increase of cardiovascular death.

**
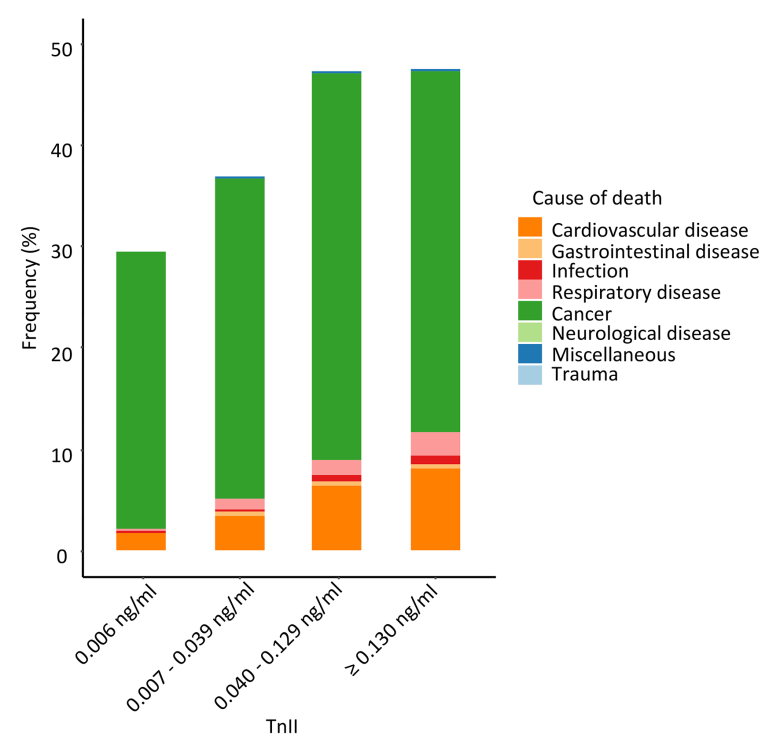
**

**Supplementary Table 1. Clinical outcome according to the cardiac troponin I (TnI) level**

|  | TnI | HR (95% CI) | p-value |
| --- | --- | --- | --- |
| All-cause death | 0.006 ng/ml | Reference | - |
|  | 0.007 - 0.039 ng/ml | 1.339 (1.238 - 1.449) | <0.001 |
|  | 0.040 - 0.129 ng/ml | 1.962 (1.755 - 2.193) | <0.001 |
|  | ≥ 0.130 ng/ml | 1.992 (1.758 - 2.256) | <0.001 |
| Cardiovascular death | 0.006 ng/ml | Reference | - |
|  | 0.007 - 0.039 ng/ml | 2.076 (1.305 - 3.303) | <0.001 |
|  | 0.040 - 0.129 ng/ml | 5.633 (3.362 - 9.439) | <0.001 |
|  | ≥ 0.130 ng/ml | 9.301 (5.696 - 15.189) | <0.001 |
| Non-cardiovascular death | 0.006 ng/ml | Reference | - |
|  | 0.007 - 0.039 ng/ml | 1.318 (1.216 - 1.428) | <0.001 |
|  | 0.040 - 0.129 ng/ml | 1.872 (1.669 - 2.100) | <0.001 |
|  | ≥ 0.130 ng/ml | 1.820 (1.596 - 2.075) | <0.001 |
